# Supplementary material for: Photocatalytic dehydrogenative C-C coupling of acetonitrile to succinonitrile
Source: Nat Commun. 2022 Jul 28;13:4379. doi: 10.1038/s41467-022-32137-y (PMC9334571; doi:10.1038/s41467-022-32137-y)
Supplement: Supplementary file 1 — Supplementary Information [file 41467_2022_32137_MOESM1_ESM.pdf]

Supporting Information

Photocatalytic Dehydrogenative C-C Coupling of Acetonitrile to Succinonitrile

Xian Zhou,[1] Xiaofeng Gao,[2] Mingjie Liu,[1] Zirui Gao,[2] Xuetao Qin,[2] Wenhao Xu,[1] Shitong Ye,[1] Wenhua Zhou,[1] Haoan Fan,[1] Jing Li,[1] Shurui Fan,[3] Lei Yang,[4] Jie Fu,[1] Dequan Xiao,[5] Lili Lin,[3*] Ding Ma,[2*] and Siyu Yao,[1,2*]

* corresponding author

[1] Key Laboratory of Biomass Chemical Engineering of Ministry of Education, College of Chemical and Biological Engineering, Zhejiang University, Hangzhou 310027, China.

[2] Beijing National Laboratory for Molecular Sciences, College of Chemistry and Molecular Engineering and College of Engineering, Peking University, Beijing, China.

[3] Institute of Industrial Catalysis, State Key Laboratory of Green Chemistry Synthesis Technology, College of Chemical Engineering, Zhejiang University of Technology, Hangzhou 310014, Zhejiang, China.

[4] Zhejiang Henglan Science and Technology Co. Ltd, Hangzhou 310027, China.

[5] Center for Integrative Materials Discovery, Department of Chemistry and Chemical and Biomedical Engineering, University of New Haven, West Haven, CT 06516, USA.

Experimental Section

Materials.

All chemicals were used directly without further purification. Anatase TiO_2 (TiO_2-A) and rutile TiO_2(TiO_2-R) were purchased from Aladdin. TiO_2(P25) (TiO_2-P), which contained 25% rutile and 75% anatase, was purchased from Degussa. $H_2PtCl_6\cdot6H_2O$, $HAuCl_4\cdot4H_2O$, $PdCl_2$, $RhCl_3\cdot3H_2O$, $AgNO_3$, and $Fe(NO_3)_3\cdot9H_2O$ were purchased from Macklin.

Catalyst Preparation.

The M/TiO_2 catalysts were prepared by photodeposition method.[1] In the photodeposition (PD) of metal over TiO_2 support, 200mg TiO_2 powder and metal precursor ($H_2PtCl_6\cdot6H_2O$ / $HAuCl_4\cdot4H_2O$ / $PdCl_2$ / $RhCl_3\cdot3H_2O$ / $AgNO_3$) were dispersed in 15 mL 20% methanol aqueous solution (v/v) in a quartz flask. Subsequently, the flask was placed in the 365 nm photo-radiation generated by a 1W LED lamp under argon protection with vigorous stirring for 3 h. After the PD procedure, the M/TiO_2 was separated from the suspension by centrifugation, washed 3 times using DI water and dried at 333 K for 12 h before use.

The other Pt modified semiconductor catalysts were prepared using similar procedure as the M/TiO_2 photocatalysts.

Sample Characterizations.

ICP-AES

The loadings of supported metals on the catalysts were analyzed by inductively coupled plasma atomic emission spectrometry (ICP-AES) on a Varian ICP-OES 720 instrument. In a typical procedure, 25mg catalyst powder was dissolved by 3 mL HF and 4 mL chlorazotic acid. The solution as filtrated and diluted by DI water for further measurement.

X-ray Diffraction characterization

Powder X-ray diffraction (XRD) patterns were recorded in the 2θ range 10°-80° on a Philips X'Pert Pro Super diffractometer with Cu Kα radiation (λ = 0.15406 nm) operating at 40 kV and 50 mA.

X-ray absorption spectroscopic (XAS) characterization

The Pt L_3 edge XAS characterization was performed at BL-14W beamline of Shanghai synchrotron radiation facility (SSRF) under fluorescence mode. The fine powder of catalysts was dispersed on Kapton

tape and folded to get sufficient signals. The data pretreatment and fitting of EXAFS spectra were performed using Ifeffit packages[2].

Transmission electron microscopy (TEM)

The TEM and HADDF-STEM images of catalysts were performed with a JEOL JEM-2100F instrument at an acceleration voltage of 200 kV.

Electron paramagnetic resonance (EPR) spectra

Electron paramagnetic resonance (EPR) spectra were recorded on a JEOL JES-FA200 EPR spectrometer (9.063 GHz, X-band) at 130 K with employed microwave power, modulation frequency, and modulation amplitude of 0.998 mW, 100 kHz, and 0.35 mT, respectively.

UV-Vis Spectroscopic characterization

UV-vis diffuse reflectance spectra (UV-vis DRS) were obtained on a Shimadzu DUV-3700 spectrophotometer equipped with an integrating sphere attachment.

Transient photocurrent [3]

The 5mg sample was mixed with 0.5ml ethanol solution for ultrasonic treatment for 1 hour, and then the prepared sample was dropped onto ITO conductive glass of 1 cm × 2 cm. The sample was dried in the oven at 60℃ to ensure that the sample would not fall off. The glass was used as the working electrode. The electrolyte solution was 1 M Na_2SO_4 solution with reference electrode of mercurous sulfate and counter electrode of Pt. The electrochemical workstation was manufactured by Shanghai Chenhua Instrument Co., LTD. The test method was i-t Curve with a sensitivity of 1.e-6. During the test, the conductive glass stained with the sample was inserted into the electrolyte solution and fixed in a suitable position. Then turn on the light (with a 365nm filter) and test for 20s. Repeat the above steps 5 times to obtain the photocurrent spectrum.

Photoluminescence spectroscopy

Horibalabram-hr confocal laser microraman spectrometer produced by JY, France, was used for PL spectrum. The excitation light source was HE-Cd laser (365nm) and the grating was 2400.

Fluorescence probe experiment

Photocatalyst powder (10 mg) was suspended in a Pyrex cell containing 10 mL of a 0.1 mM coumarin aqueous-CH_3CN solution, and one side of the cell was irradiated with the LED beam. The suspension was

stirred vigorously for 20 min before and during the light irradiation at 60 °C. After the irradiation, the clear solution was taken out and its fluorescence spectrum was measured by the Fls1000 fluorescence spectrophotometer with the excitation wavelength at 332 nm.

Photocatalytic Reaction Test.

Photocatalytic experiments (acetonitrile dehydrogenative coupling to succinonitrile) were performed in a top-irradiation Pyrex flask. A 10W LED light (wavelength 365 nm) (PLS-SXE300, Beijing Trusttech Co., Ltd.) was used as the light source. Typically, 20 mg photocatalysts were dispersed in 10 mL 70% volume acetonitrile aqueous solution under magnetic stirring. Prior to the irradiation, the reaction mixture was deaerated repeatedly with Ar gas for 5 times to thoroughly remove air and dissolved oxygen. During the reaction, the photocatalytic reaction system was kept at 60°C. To evaluate the photocatalytic hydrogen production and analyze other gas products, the gas-phase composition of the photocatalytic reactor was analyzed by an Agilent 8860 gas chromatograph equipped with 5 Å molecular sieves and HP-Plot columns and thermal conductivity cell (TCD) detector. Liquid products were analyzed by Agilent 8860 gas chromatograph equipped with a column of SH-1 with flame ionization detector (FID). The formation rate of all products, average turnover frequency (ATOF) and carbon-based selectivity of products were defined in equation (1) - (3).

Formation rate

$$r=\frac{n_x}{t\times m_{cat}} \qquad \text{Eq (1)}$$

Average turnover frequency

$$ATOF=\frac{1}{N_{AS}}\frac{dn}{dt} \qquad (N_{AS}\text{: The number of Pt atoms in the catalyst determined by ICP-AES}) \qquad \text{Eq (2)}$$

Carbon based selectivity of products

$$S_x=\frac{a\times n_x}{4\times n_{SN}+n_{CO2}+2\times n_{AM}} \qquad (\text{a: The number of C atoms in x molecule}) \qquad \text{Eq (3)}$$

AQY calculation methods.

In generally, producing one $\cdot CH_2CN$ radical from CH_3CN needs one hole with one electron consumed at the same time (e.g., one SN molecule needs two electrons). Then the AQY could be calculated according to the following Equation:

$$\text{AQY}(\%) = \frac{\text{number of reacted electrons}}{\text{number of incident photons}} \times 100\%$$

$$= \frac{\text{number of evolved SN molecules} \times 2}{\text{number of incident photons}} \times 100\% \qquad \text{Eq (4)}$$

The total light intensity incident (305 mW/cm^2) at the sample position is measured by using CEL-NP2000-2A flux meter. Assuming uniform intensity distribution of the lamp, a correction for the difference in the area of the sensor of lux meter and the reactor surface area is evaluated.

AQY calculation:

Number of incident photons:

$$\text{N} = \frac{I \times A \times \lambda \times t}{h \times c} = \frac{3.05 \times 10^{3} \times 1.77 \times 10^{-4} \times 365 \times 10^{-9} \times 3600}{6.626 \times 10^{-34} \times 3 \times 10^{8}} = 3.57 \times 10^{21} \qquad \text{Eq (5)}$$

I: optical power density (W·m^{-2})

A: incident illumination area (m^2)

λ : wavelength of incident light (m)

t: time (s)

H: Planck constant (6.626×10^{-34} J·s)

C: the speed of light (3×10^8 m·s^{-1})

$$\text{AQY}(\%) = \frac{\text{number of evolved SN molecules} \times 2}{\text{N}} \times 100\%$$

$$= \frac{N_A \times n \times 2}{N} \times 100\% = \frac{6.02 \times 10^{23} \times 1.31 \times 10^{-4} \times 2}{3.57 \times 10^{21}} \times 100\% = 4.4\% \qquad \text{Eq (6)}$$

N_A: Avogadro's constant (6.02×10^{23} mol^{-1})

n: moles of SN per hour

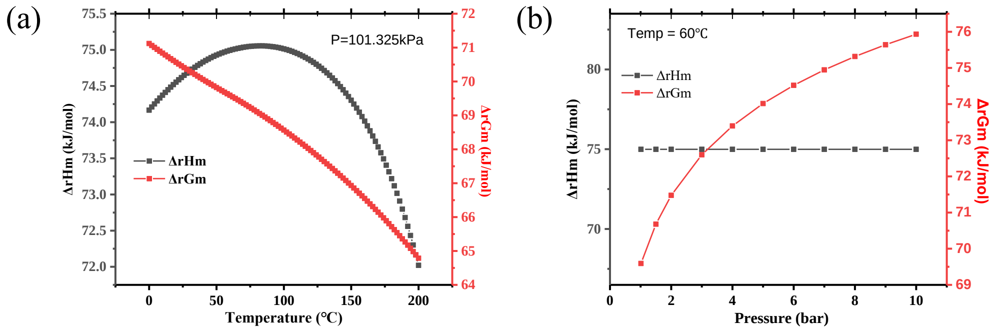

Supplementary Figure 1. Enthalpy and Gibbs free energy changes for the dehydrogenative coupling of acetonitrile to succinonitrile, (a) Influence of temperature at constant pressure (P=101.325 kPa); (b) Influence of system pressure at constant temperature (T=60°C).

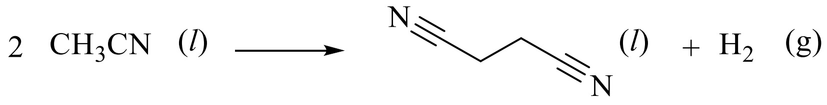

Δ_rHm (60℃,101kPa) = 75.0 kJ/mol

Δ_rGm (60℃,101kPa) = 69.6 kJ/mol

Δ_rGm (60℃,301kPa) = 72.6 kJ/mol

Δ_rGm (150℃,301kPa) = 70.7 kJ/mol

The thermodynamic evaluation of the CH_3CN dehydrogenative coupling reaction in Figure S1 suggests that the dehydrogenative coupling of acetonitrile is thermodynamically unfavourable reaction, which cannot occur spontaneously at ambient condition.

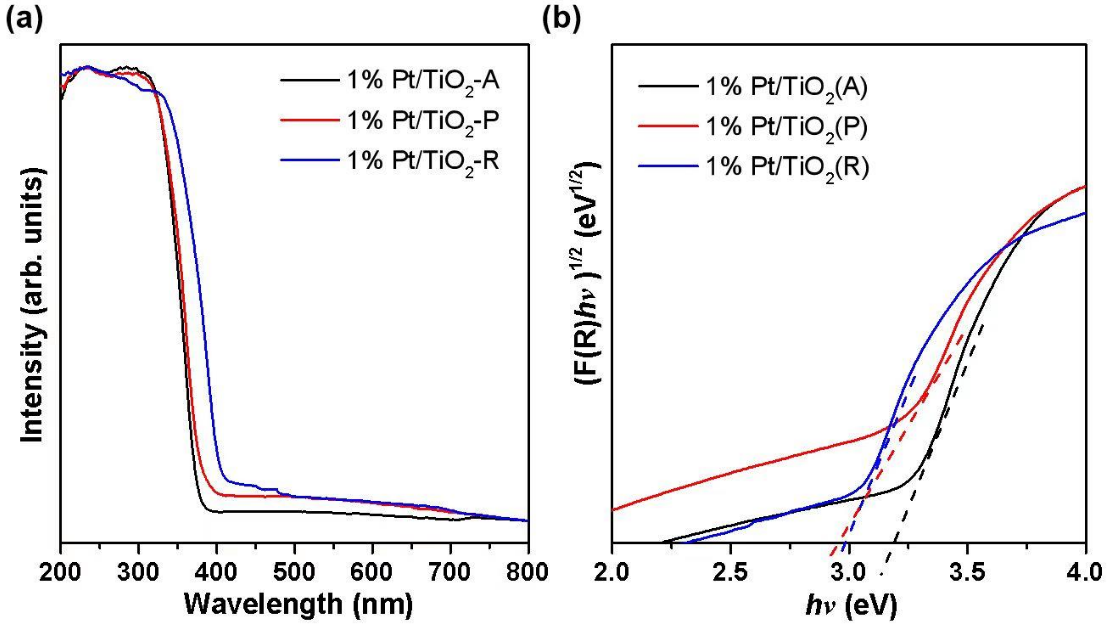

Supplementary Figure 2. (a) UV-Vis diffuse reflectance spectra of various 1% Pt/TiO_2 photocatalysts; (b) The plots of modified Kubelka-Munk function versus the photon energy of various 1% Pt/TiO_2 photocatalysts.

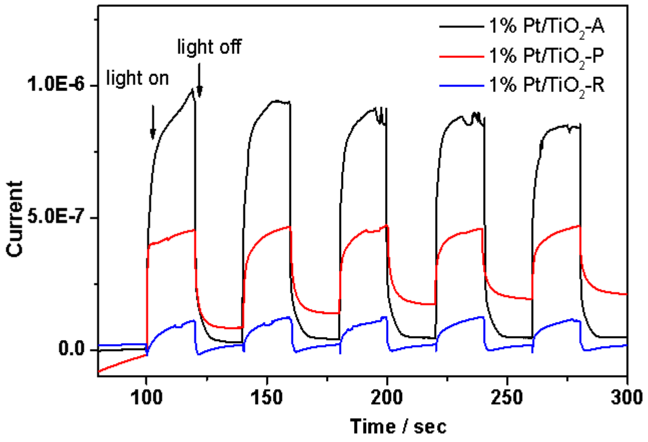

Supplementary Figure 3. Transient photocurrent responses for various 1% Pt/TiO_2

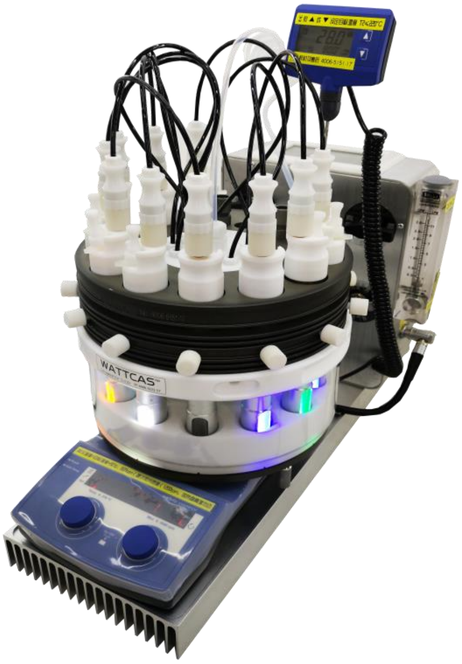

Supplementary Figure 4. Photocatalytic reactor used for the catalyst evaluation for the CH_3CN dehydrogenative coupling reaction.

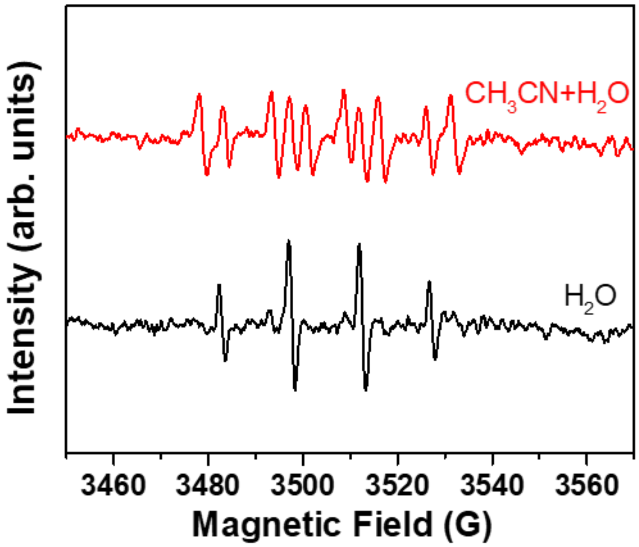

Supplementary Figure 5. *In situ* electron paramagnetic resonance (EPR) spectra for systems containing Pt/TiO_2-A in acetonitrile aqueous solution or pure water solution in the presence of DMPO (a spin-trapping agent) with light irradiation.

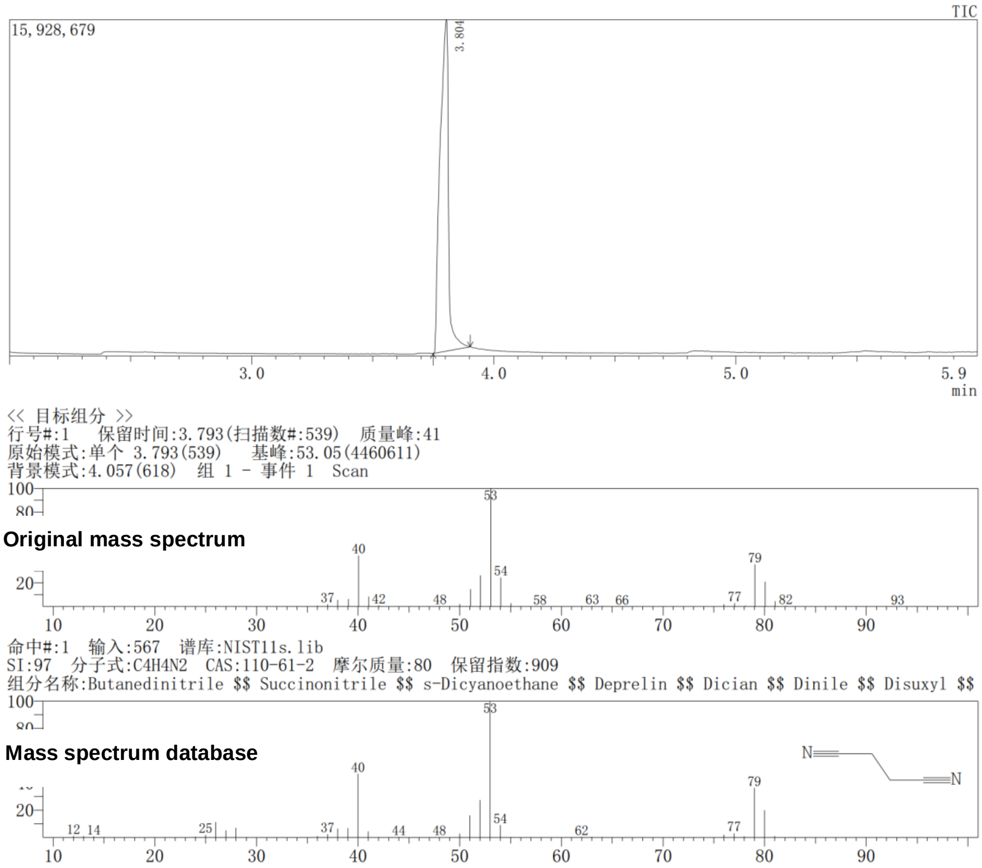

Supplementary Figure 6. GC-MS analysis of the product succinonitrile.

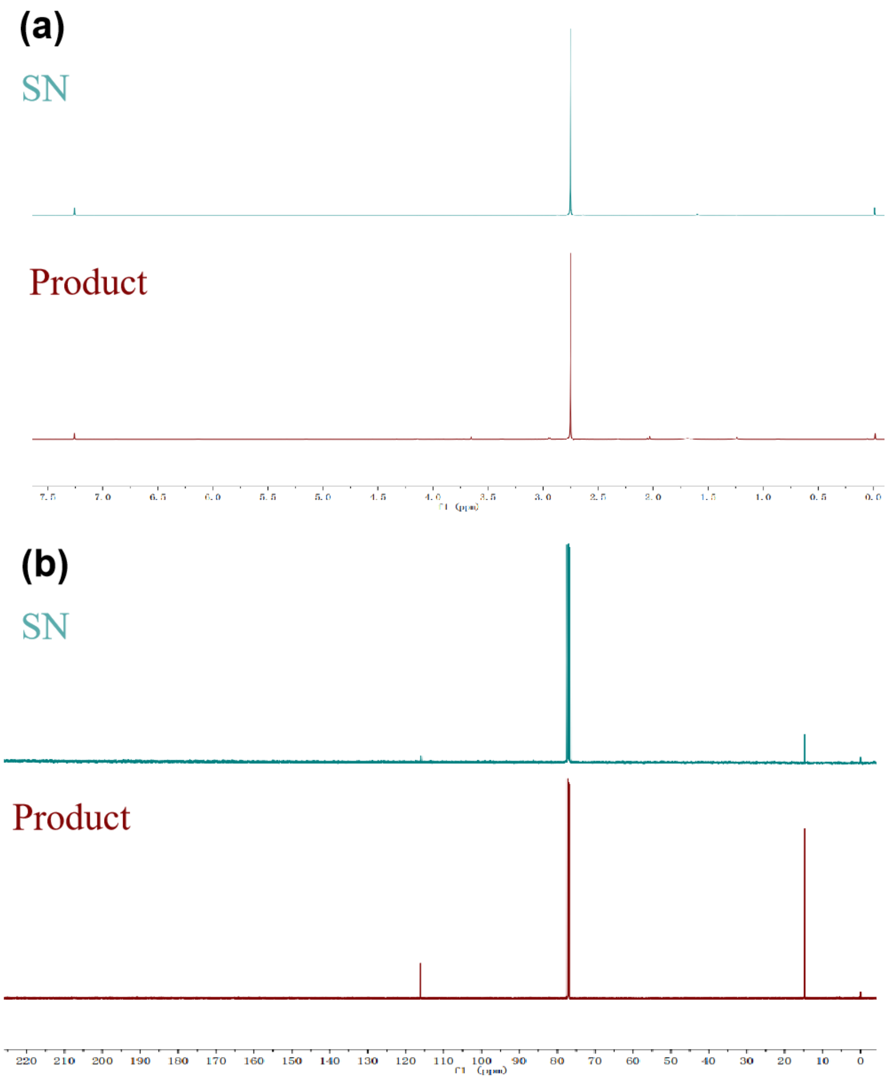

Supplementary Figure 7. NMR spectra of succinonitrile standard sample and product, (a) ^{1}H NMR spectra; (b) ^{13}C NMR spectra.

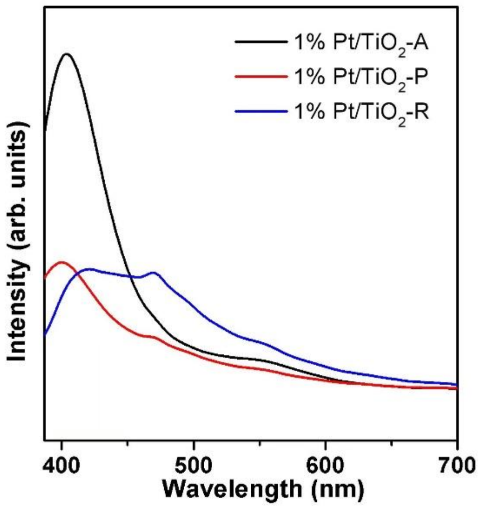

Supplementary Figure 8. Photoluminescence spectra of 1% Pt/TiO_2 catalysts.

Supplementary Discussion

The photoluminescence spectra in Supplementary Figure 8 was used to understand the electron separation behaviors of Pt/TiO_2 catalysts and explain the catalytic performances. The emission peak around 400 nm arises due to self-trapped excitons (STE) localized on TiO_6 octahedra or to an oxygen vacancy with two trapped electrons. The STE can be created by direct recombination of a trapped electron on the lattice site with a hole or the by indirect recombination assisted by an oxygen vacancy. The emission signals around 468 nm and 552 nm can be assigned to the charge-transfer from Ti^{3+} to oxygen anion in a TiO_6 octahedron associated with oxygen vacancies. The emission around 400 nm may originate from oxygen vacancies that form shallow traps near the surface of the nanocrystals, while the emission at 552 nm is associated with oxygen vacancies that form deep traps. It is seen that there exists a direct correlation between the intensity ratio of the emission peaks from shallow and deep traps and the photocatalytic activity of the samples. The emission intensity at 400 nm is found to be the maximum for the sample 1% Pt/TiO_2(A) which exhibited the highest photocatalytic activity. From our analysis, it can be inferred that the shallow trap states would have helped in the effective separation of the charge carriers to facilitate efficient photocatalytic activity. Deep trap centers, on the other hand, favor recombination of charge carriers and hinder the photocatalytic activity. The emission intensity of 1% Pt/TiO_2(R) is found to be the maximum at 552 nm while minimum at 416 nm, which exhibited the lowest photocatalytic activity.

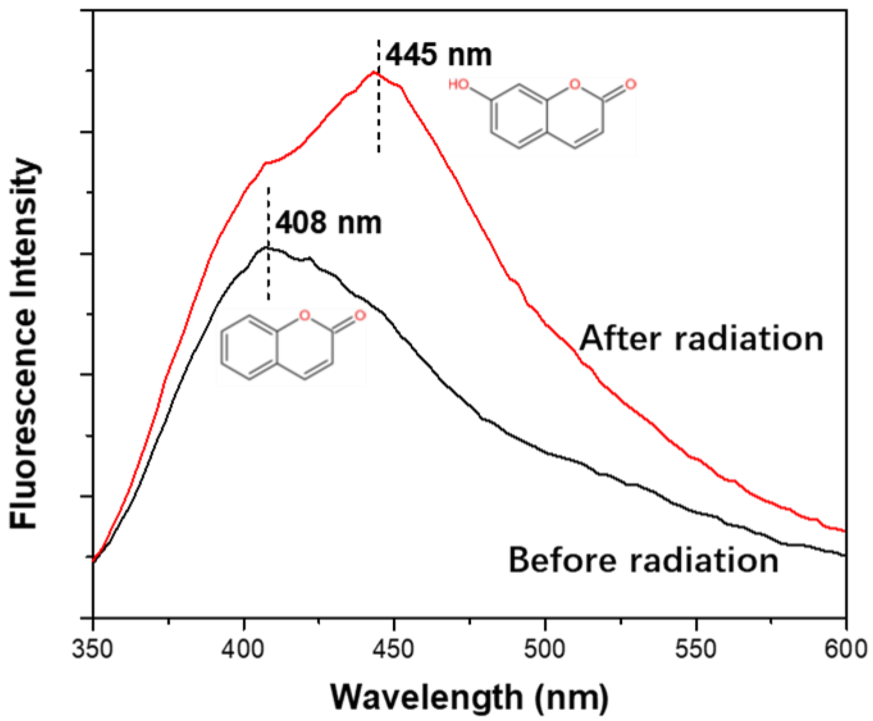

Supplementary Figure 9. Fluorescence probe method to detect the existence of ·OH radical in solution.

Supplementary Discussion

The fluorescence probe method was used to detect the hydroxyl radical desorbed from the catalyst surface after radiation. The coumarin (408 nm) was used as the probe which would capture the free ·OH and transform into umbelliferone (445nm). Significant amount of free ·OH radicals was observed after 20 min radiation at 60 °C, suggesting the coupling reaction occurred both in the solution and on the surface of catalysts.

Supplementary Table 1. Pt L_3 edge EXAFS fitting results of 1% Pt/TiO_2-A, -P and -R catalysts

Catalysts	Shell	Bond length (Å)	Coordination number	σ^2 (10^{-3} Å^2)	E_0 shift (eV)
1% Pt/TiO_2-A	Pt-Pt	2.74±0.01	9.0±0.6	4±1	5.8
1% Pt/TiO_2-P	Pt-Pt	2.74±0.02	8.9±0.9	4±1	6.0
1% Pt/TiO_2-R	Pt-Pt	2.75±0.01	9.9±1.1	5±1	6.4

Supplementary Table 2. Catalytic performance of TiO_2 based photocatalysts

Catalyst [a]	Formation rate (mmol g_{cat}^{-1} h^{-1})				Carbon based Selectivity (%) [b]			TOF (h^{-1}) [c]	AQY (%)
	SN [d]	H_2	CO_2	AM [e]	SN	CO_2	AM		
TiO_2-R	0	0	0	0	0	0	0	0	0
TiO_2-P	0	0.03	0	0.32	0	0	100	0	0
TiO_2-A	0	0.04	0	0.35	0	0	100	0	0
Pt/TiO_2-R	0	0.17	0.08	0	0	100	0	0	0
Pt/TiO_2-P	4.97	9.03	0.31	0	98.5	1.5	0	108	3.3
Pt/TiO_2-A	6.55	9.76	0.38	0.15	97.5	1.4	1.1	142	4.4
Au/TiO_2-A	1.26	2.55	0.12	0.12	93.3	2.2	4.5	28	0.8
Rh/TiO_2-A	1.28	1.97	0.19	0.03	95.3	3.5	1.2	14	0.9
Pd/TiO_2-A	0.46	0.69	0.32	0.15	74.8	13.0	12.2	5	0.3
Ag/TiO_2-A	0.32	0.08	0.17	32.02	2.0	0.3	97.7	3	0.2
Fe/TiO_2-A	0.29	0	0.43	0.02	71.2	26.4	2.4	2	0.2

a. Reaction condition: Solution: 7mL CH_3CN + 3mL H_2O; Atmosphere: Ar; Light source: 10W LED lamp; UV-Vis light, λ = 365 nm; Irradiation time: 2 h

b. Carbon based selectivity is determined by GC.

c. Turnover frequency of the catalyst is defined as the average formation rate of SN per unit metal per hour.

d. Succinonitrile; **e.** Acetamide

Supplementary Table 3. Catalytic performance of various Pt/Semiconductor based photocatalysts

Catalyst [a]	Formation rate (mmol g_{cat}^{-1} h^{-1})				Carbon based Selectivity (%) [b]			TOF (h^{-1}) [c]	AQY (%)
	SN [d]	H_2	CO_2	AM [e]	SN	CO_2	AM		
Pt/$SrTiO_3$	0	0.19	0.15	0	0	100	0	0	0
Pt/g-C_3N_4	0	0.44	0.25	0	0	100	0	0	0
Pt/ZnO	0.02	0	1.49	0	5	95	0	0.4	0
Pt/CdS	0	0	0.2	0	0	100	0	0	0
Pt/$Zn_2In_2S_5$	0	0	0	0	0	0	0	0	0
Pt/Bi_2MoO_6	0.22	0.05	0	0	100	0	0	2	0.02
Pt/BiOCl	0.16	0.36	0.11	0	85	15	0	1.5	0.01

a. Reaction condition: Solution: 7mL CH_3CN + 3mL H_2O; Atmosphere: Ar; Light source: 10W LED lamp; UV-Vis light, λ = 365 nm; Irradiation time: 2 h

b. Carbon based selectivity is determined by GC.

c. Turnover frequency of the catalyst is defined as the average formation rate of SN per unit metal per hour.

d. Succinonitrile;

e. Acetamide

Supplementary Table 4. Optimization of the reaction conditions and control experiments

Reaction conditions	Formation rate (mmol g_{cat}^{-1} h^{-1})				Carbon based Selectivity (%)			ATOF (h^{-1})	Amount of SN (mmol)
	SN	H_2	CO_2	AM	SN	CO_2	AM		
**The ratio of CH_3CN and H_2O**									
10:0	0.26	0.10	2.08	0	33.3	66.7	0	6	0.01
9:1	5.76	8.49	0.57	0.10	96.8	2.4	0.8	125	0.23
7:3	6.55	9.76	0.38	0.15	97.5	1.4	1.1	142	0.26
5:5	6.45	8.39	0.47	0.25	96.4	1.7	1.9	140	0.26
3:7	3.78	7.08	0.72	0.24	92.6	4.4	3.0	82	0.15
**Amount of the catalyst**									
5 mg	2.61	1.79	0.23	0.05	96.9	2.1	1.0	58	0.03
10 mg	3.57	6.01	0.35	0.10	96.3	2.4	1.3	72	0.07
20 mg	6.55	9.76	0.38	0.15	97.5	1.4	1.1	142	0.26
30 mg	6.28	7.04	0.39	0.20	97.0	1.5	1.5	136	0.38
**Reaction time**									
0.5 h	9.01	17.48	1.16	0.40	94.8	3.1	2.1	195	0.09
1.0 h	7.46	11.84	0.60	0.34	95.9	1.9	2.2	162	0.15
2.0 h	6.55	9.76	0.38	0.15	97.5	1.4	1.1	142	0.26
4.0 h	4.65	7.32	0.24	0.09	97.8	1.3	0.9	101	0.37
6.0 h	4.45	3.74	0.18	0	99.0	1.0	0.0	96	0.53
**Reaction Cycles**									
1	6.55	9.76	0.38	0.15	97.5	1.4	1.1	142	0.26
2	4.91	7.59	0.29	0.11	97.5	1.4	1.1	106	0.19
3	4.41	7.45	0.34	0.12	96.8	1.9	1.3	96	0.18
4	3.42	7.27	0.32	0.15	95.7	2.2	2.1	71	0.14

Supplementary Table 5. Isotopic experiments for the direct photocatalytic acetonitrile coupling with the 1%Pt/TiO_2−A photocatalyst

Entry	Reactants		SN Formation rate (mmol g_{cat}^{-1} h^{-1})	k_H/k_D
1	CH_3CN	H_2O	6.55	-
2	CH_3CN	D_2O	4.03	1.6
3	CD_3CN	H_2O	1.54	4.3
4	CD_3CN	D_2O	0.85	7.7

All reaction conditions were the same as those shown in Table S2

k_H/k_D=[amount of succinonitrile in the reaction without any isotopic reactant (mmol)] /[amount of succinonitrile with a isotopic reactant (mmol)]

Supplementary Table 6. GC-MS diagram of other coupled products

Reactant	Production	GCMS
n-Butyronitrile	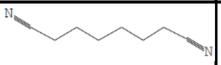	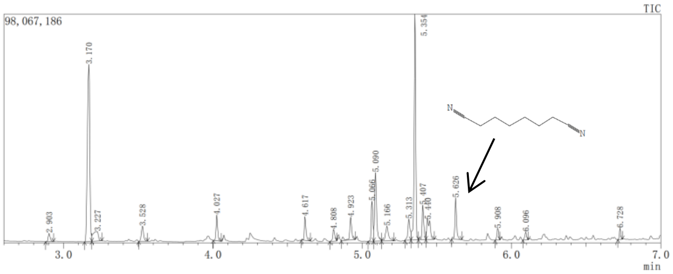
Isobutyronitrile	None	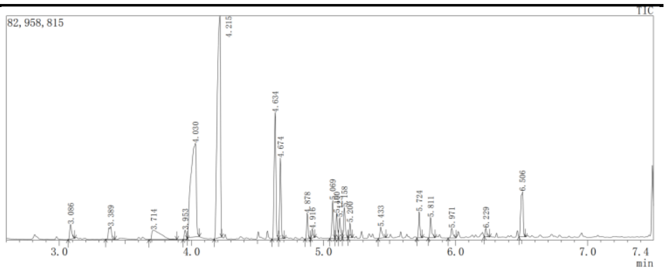
Dichloro acetonitrile	None	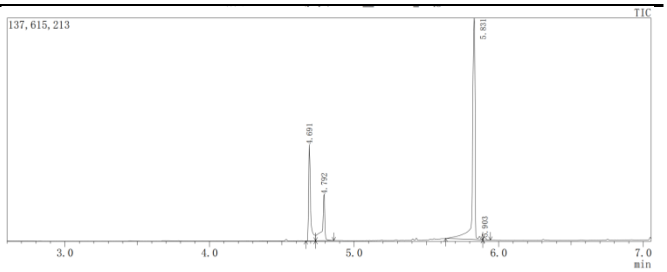
Methyl acetate	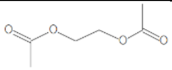	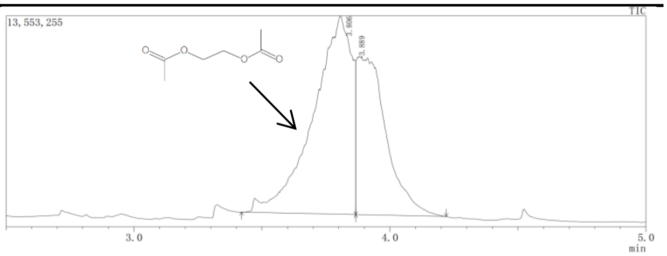
Methanol	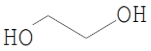	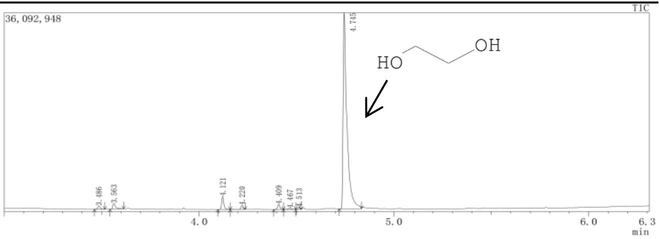

Supplementary References

1. A. Tanaka, K. Hashimoto and H. Kominami, Preparation of Au/CeO_2 Exhibiting Strong Surface Plasmon Resonance Effective for Selective or Chemoselective Oxidation of Alcohols to Aldehydes or Ketones in Aqueous Suspensions under Irradiation by Green Light. *J. Am. Chem. Soc*. **134**, 14526-14533 (2012).
2. B. Ravel and M. Newville, ATHENA, ARTEMIS, HEPHAESTUS: data analysis for X-ray absorption spectroscopy using IFEFFIT. *J. Synchrotron Rad.* **12**, 537-541 (2005)
3. S. Xie, Z. Shen, J. Deng, et al. Visible light-driven C−H activation and C–C coupling of methanol into ethylene glycol. *Nat. Commun.* **9**, 1-7 (2018).
